# CCL2/EGF positive feedback loop between cancer cells and macrophages promotes cell migration and invasion in head and neck squamous cell carcinoma

**DOI:** 10.18632/oncotarget.13523

**Published:** 2016-11-23

**Authors:** Lu Gao, Feng-qin Wang, Hui-min Li, Jie-gang Yang, Jian-Gang Ren, Ke-fei He, Bing Liu, Wei Zhang, Yi-Fang Zhao

**Affiliations:** ^1^ The State Key Laboratory Breeding Base of Basic Science of Stomatology and Key Laboratory of Oral Biomedicine Ministry of Education, School and Hospital of Stomatology, Wuhan University, Wuhan 430079, China; ^2^ Department of Oral and Maxillofacial Surgery, School and Hospital of Stomatology, Wuhan University, Wuhan 430079, China; ^3^ College of Stomatology, Dalian Medical University, Dalian 116044, China; ^4^ Department of Stomatology, The Central Hospital of Wuhan, Tongji Medical College, Huazhong University of Science and Technology, Wuhan 430014, China

**Keywords:** HNSCC, tumor associated macrophages, invadopodia, CCL2, EGF

## Abstract

Head and neck squamous cell carcinoma (HNSCC) represents the most frequent malignancy in the head and neck region, and the survival rate has not been improved significantly over the past three decades. It has been reported the infiltrated macrophages contribute to the malignant progression of HNSCC. However, the crosstalk between macrophages and cancer cells remains poorly understood. In the present study, we explored interactions between monocytes/macrophages and HNSCC cells by establishing the direct co-culture system, and found that the crosstalk promoted the migration and invasion of cancer cells by enhancing the invadopodia formation through a CCL2/EGF positive feedback loop. Our results demonstrated HNSCC cells educated monocytes into M2-like macrophages by releasing C-C motif chemokine ligand 2 (CCL2, or MCP-1). And the M2-like macrophages secreted epithelial growth factor (EGF), which increased the motility of HNSCC cells by enhancing the invadopodia formation. These subcellular pseudopodia degraded extracellular matrix (ECM), facilitating tumor local invasion and distant metastasis. Moreover, EGF up-regulated CCL2 expression in HNSCC cells, which recruited monocytes and turned them into M2-like macrophages, thus forming a positive feedback paracrine loop. Finally, we reported that curcumin, a powerful natural drug, suppressed the production of EGF and CCL2 in macrophages and cancer cells, respectively, blocking the feedback loop and suppressing the migration and invasion of HNSCC cells. These results shed light on the possibilities and approaches based on targeting the crosstalk between cancer cells and monocytes/macrophages in HNSCC for potential cancer therapy.

## INTRODUCTION

Head and neck squamous cell carcinoma (HNSCC) is one of the most frequent cancers in the world. Despite the rapid development of diagnostic and therapeutic approaches in recent years, the 5-year survival rate of patients suffering HNSCC is still less than 50%, which remains over the past three decades [1, 2]. The local recurrence and distant metastases are considered to be the major reasons for the poor prognosis [3]. Thus, the underlying mechanisms for the local invasion and distant metastases of HNSCC need to be better understood.

Persistent inflammation induced by smoking, excessive alcohol consumption, repetitive injury and human papillomavirus (HPV) infection has been evidenced as the critical factor during the initiation and development of most HNSCCs [4, 5]. The infiltrated macrophages in tumors (termed as tumor associated macrophages, TAMs), as the major inflammatory cellular population in the tumor microenvironment, promote the progression of malignancies by supporting tumor growth, enhancing tumor associated angiogenesis, suppressing adaptive immunity, and assisting tumor cells in invading into surrounding normal tissues and metastasizing to distant organs [6]. Monocytes enter tumor tissues through blood vessels, and then differentiate into macrophages under the stimuli from tumor cells or the tumor associated microenvironment [7]. Accumulating research has demonstrated the crosstalk between tumor cells and macrophages (or monocytes) contributed to the recruitment of macrophages in various cancers, including breast cancer, lung cancer, hepatocellular carcinoma, *etc*. [8–10]. However, the interactions between monocytes/macrophages and HNSCC cells, as well as the consequent effects on the progression of HNSCC are largely unknown.

Efficient tumor invasion and metastasis through tissue barriers or blood/lymphatic vessel walls require potent ability of extracellular matrix (ECM) degradation [11]. Recent studies have reported the specialized subcellular structures, which were named as invadopodia in malignant cells and podosomes in many normal cell types, including vascular endothelial cells, macrophages, osteoclasts, *etc*., involved in the degradation of ECM [12, 13]. These membrane protrusions facilitate cancer cells penetration through the basement membrane of blood vessels and into surrounding tissues, therefore promoting local invasion and distant metastasis of the cancer cells. Our previous study demonstrated that the infiltration of TAMs in cancer tissues was successively increased compared with that in the normal tissues. The number of infiltrated macrophages was strongly associated with lymph node metastasis [14]. However, whether the infiltrated macrophages regulate the formation of invadopodia and then promote the migration and invasion of HNSCC cells are still unclear.

We here for the first time performed a detailed analysis of the sequential steps involved in the crosstalk between monocytes and HNSCC cells, as well as the consequential effects on the motility of cancer cells. Our data revealed that HNSCC cells recruited and educated monocytes into M2- polarized macrophages, which in turn promoted the migration and invasion of the cancer cells by enhancing the their invadopodia formation in an EGF dependent manner. And we found that the EGF/EGFR-CCL2/CCR signaling pathways in the co-culture system constituted a positive feedback paracrine loop. Finally, we tested and found that curcumin, a natural polyphenolic compound derived from the root of *Curcuma longa*, suppressed the expression of CCL2 in HNSCC cells and EGF in macrophages, weakened the invadopodia formation and alleviated HNSCC cells migration and invasion, showing a promising therapeutic potential in HNSCC.

## RESULTS

### HNSCC cells educate monocytes into M2- polarized macrophages

**Figure 1:**
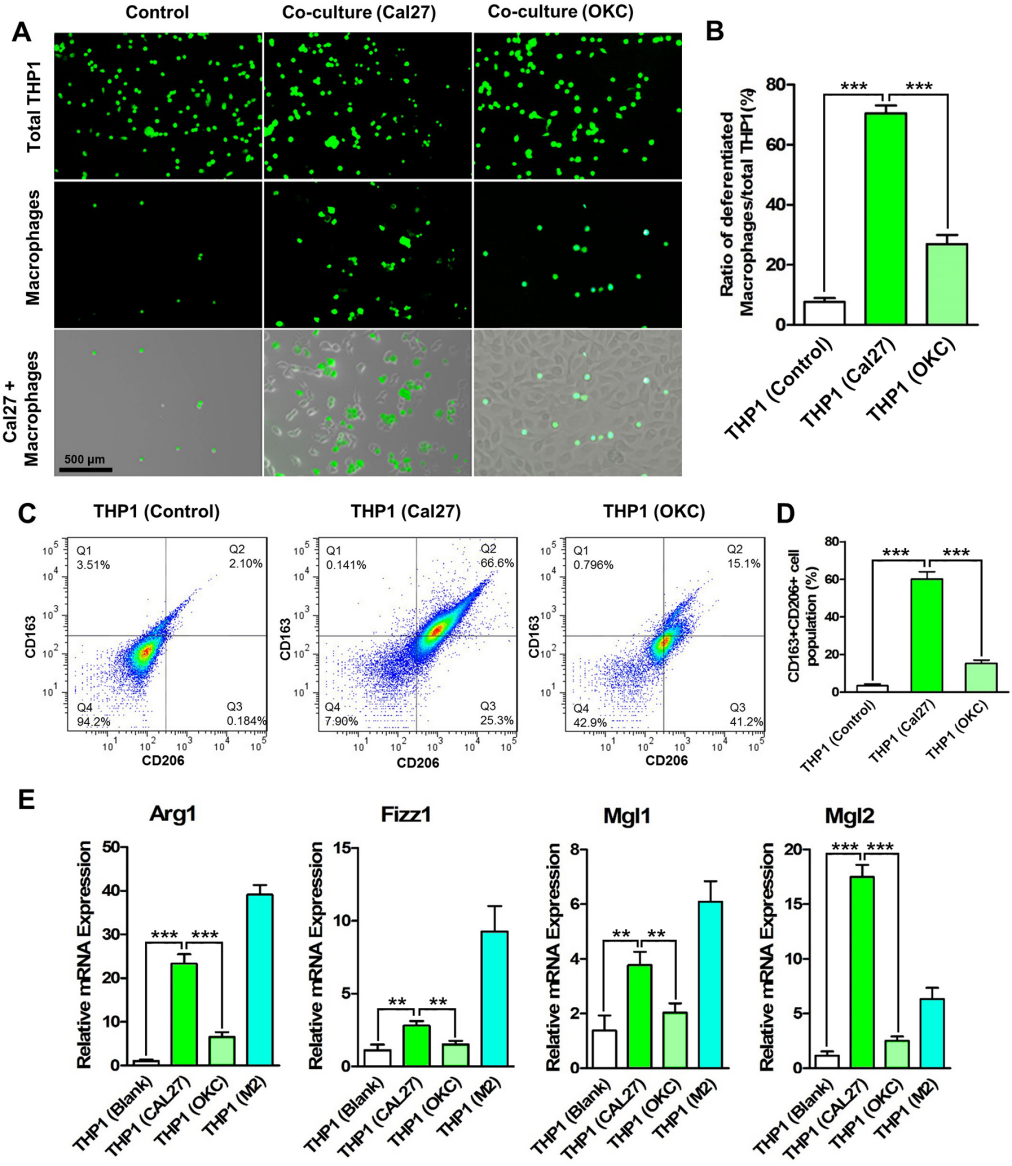
HNSCC cells promote the differentiation and M2- polarization of THP1 cells CFDA-SE labeled THP1 cells were added to the dishes in which Cal27 cells were seeded for 6 h. After co-culture for 24 h, unattached THP1 cells were washed out. (**A**) Cal27 cells promote the differentiation of THP1 cell into macrophages, most of which were attached on the cancer cells. (**B**) The ratio of attached cells to the total THP1 population was calculated. Co-culture of THP1 cells and oral keratinocytes (OKC) was established as a control. Then the CFDA-SE labeled THP1 cells were sorted by FACS. (**C**) The membrane protein expression of CD163 and CD206 was analyzed by flow cytometry. (**D**) The CD163 and CD206 double positive THP1 cells were quantitatively analyzed. After the unattached THP1 cells were washed out, the CFDA-SE labeled THP1 cells were sorted. (**E**) The mRNA expression of M2- polarized macrophage’s markers Arg1, Fizz1, Mgl1 and Mgl2 were analyzed. **P < 0.01; ***P < 0.001.

High infiltration of tumor associated macrophages in HNSCCs was reported in our previous study [14]. And a recent study has also demonstrated that TAMs could be recruited by the tumor microenvironment of HNSCC [15]. However, the precise mechanism by which monocytes are recruited is still unclear. Here, we firstly established a direct co-culture system between monocytes and HNSCC cells by culturing THP1 and Cal27 together for 24 h. THP1 cells were stained with a green fluorescent dye CFDA-SE for visualization. As shown in Figure 1A, equal amounts of THP1 cells were seeded in the blank dishes (Control group) and the dishes pre-cultured with Cal27 cells or oral keratinocytes (OKC). After co-culture for 24 h, the unattached cells were washed out, and the attached cells were observed under a microscope. The results demonstrated the number of differentiated macrophages, the adherent THP1 cells, was significantly increased when co-cultured with Cal27 cells, reaching approximate 70% of the total population (Figure 1A and 1B). Less than 10% THP1 cells were adherent on the blank dishes and approximate 30% THP1 cells were adherent when co-cultured with OKCs. Moreover, another HNSCC cell line FaDu also promoted the adherence of THP1 cells (Supplementary Figure S1). The CFDA-SE labeled THP1 cells were sorted, and analyzed by flow cytometry to quantify the membrane expression of CD163 and CD206. After co-culture with Cal27 cells, more than 60% THP1 cells displayed double positive staining of CD163 and CD206, and less than 20% THP1 cells were double positive after co-culture with OKCs (Figure 1C and 1D). Then the unattached THP1 cells were washed out, and the polarization of the macrophages was investigated by quantitatively analyzing the mRNA expression profile of the specific biomarkers using qPCR. The results demonstrated the mRNA expression levels of Arg1, Fizz1, Mgl1 and Mgl2 were significantly increased in the attached THP1 cells that were co-cultured with Cal27 cells, but not those co-cultured with OKCs (Figure 1E). THP1 cells without any treatment and M2-polarized macrophages, which were induced from THP1 cells treated with phorbol 12-myristate 13-acetate (PMA) and IL-4/IL-13 cytokines, were used as negative and positive control, respectively. All above data suggested that HNSCC cells promoted the differentiation of monocytes into macrophages, and educated them into M2- polarized phenotype.

### CCL2 contributes to the differentiation of monocytes into M2- polarized macrophages

**Figure 2:**
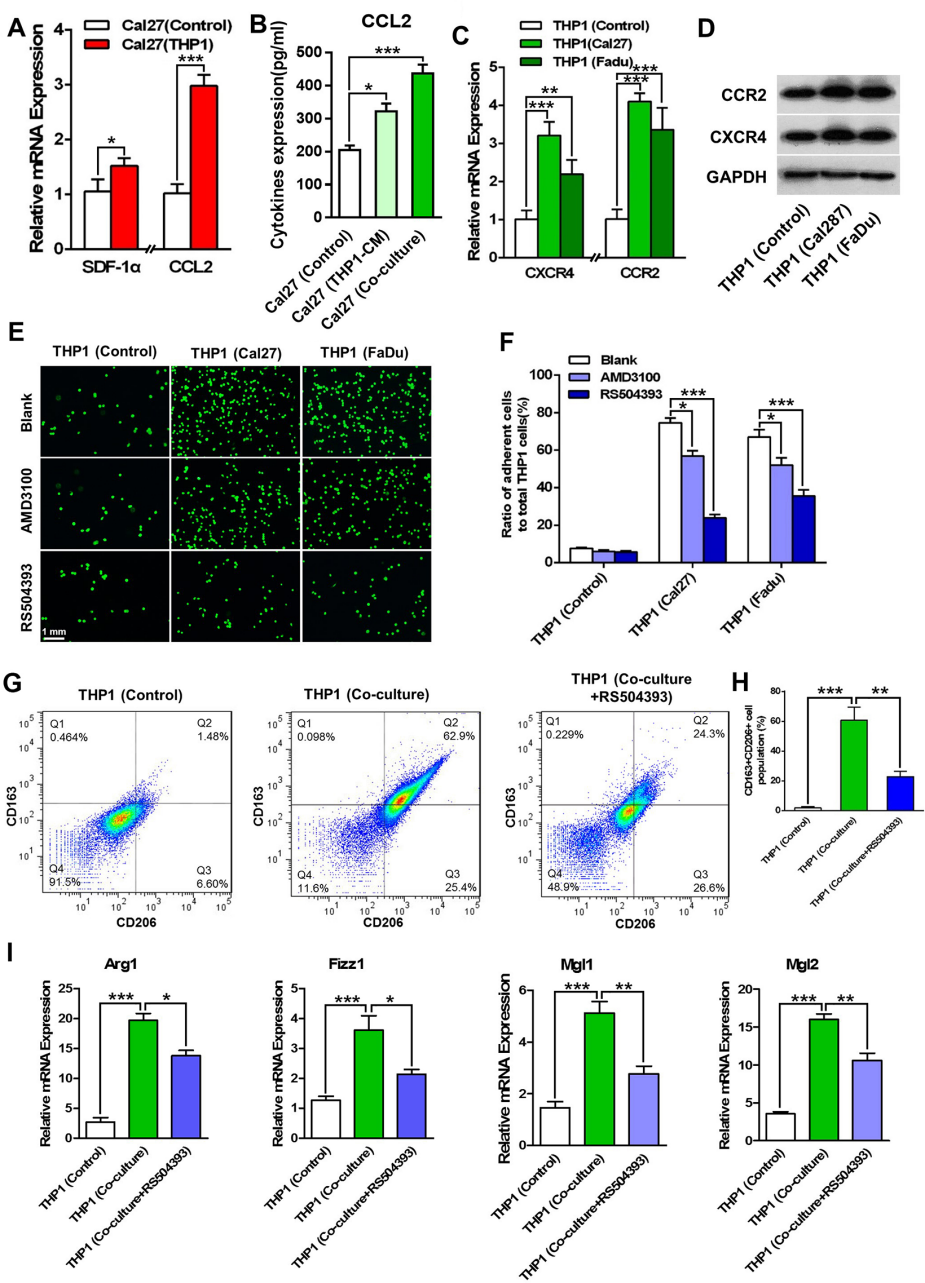
CCL2/CCR2 axis involves in the differentiation and M2- polarization of THP1 cells The sorted HNSCC cells (including Cal27 and FaDu cells) were analyzed for the expression of chemotactic factors, and meanwhile the corresponding receptors were measured in the co-cultured THP1 cells. (**A**) The mRNA expression levels of SDF-1α and CCL2 were increased in the co-cultured Cal27 cells. (**B**) ELISA assays demonstrated the increased protein level of CCL2 in the supernatant of the co-culture system. The supernatant harvested from M2- polarized macrophages was used as a control. (**C**) The mRNA expression of CXCR4 and CCR2 were measured in THP1 cells that were co-cultured with HNSCC (Cal27 and FaDu) cells. (**D**) The protein expression of CXCR4 and CCR2 were analyzed in the co-cultured THP1 cells. (**E**) CCR2 antagonist RS504393 and CXCR4 antagonist AMD3100 reduced the number of attached THP1 cells in co-culture. (**F**) Quantitative analysis of the attached THP1 cells with the treatment of RS504393 or AMD3100. (**G**) RS504393 reduced the number of CD163 and CD206 double positive THP1 cells in the co-culture system (with Cal27 cells). (**H**) The quantitative analysis of CD163+CD206+ THP1 cells after flow cytometry. (**I**) RS504393 decreased the mRNA expression of Arg1, Fizz1, Mgl1 and Mgl2 in the attached THP1 cells. *P < 0.05, **P < 0.01, ***P < 0.001.

Cal27 cells in the co-culture system were also sorted using FACS, and the cytokines expression was analyzed. As shown in Figure 2A, the mRNA expression levels of SDF-1α and CCL2 in the co-cultured Cal27 cells were significantly increased. The mRNA expression of CCL2 was twice as high as that in the control tumor cells. Moreover, secreted CCL2 in supernatant was analyzed. The protein level of CCL2 was increased from 200 pg/ml to over 400 pg/ml in the co-culture system, which was much higher than that in the supernatant of Cal27 cells that were treated with THP1 conditioned medium (Figure 2B). Moreover, CXCR4 and CCR2, the receptors of SDF-1α and CCL2, were up-regulated in the co-cultured THP1 cells (with Cal27 or FaDu cells) in both mRNA and protein levels (Figure 2C and 2D), suggesting that SDF-1α/CXCR4 and CCL2/CCR2 axes might involve in the differentiation and polarization of THP1 cells under co-culture conditions. RS504393 (10 μM), a highly selective CCR2 chemokine receptor antagonist, significantly repressed the differentiation of THP1 cells as the number of adherent monocytes was dramatically decreased. AMD3100, a specific CXCR4 antagonist, only slightly decreased the adherent monocytes number (Figure 2E and 2F). Thus, in the following experiments, CCL2/CCR2 signaling pathway was studied. Furthermore, our data revealed that in the co-culture system containing RS504393, the ratio of CD163 and CD206 double positive THP1 cells was significantly decreased, from more than 60% to approximate 30% (Figure 2G and 2H). Moreover, the mRNA expression profile of the co-cultured THP1 cells in the presence of RS504393 was analyzed. The results from qPCR showed the remarkably declined expression of Arg1, Fizz1, Mgl1 and Mgl2 in the THP1 cells after RS504393 treatment (Figure 2I).

In order to avoid the potential influence of the cancerous property of THP1 cells on the interactions between HNSCC cells and monocytes, we then isolated primary monocytes from human peripheral blood according to the previous study [16]. The direct co-culture system between peripheral blood monocytes (PBMs) and Cal27 cells was established according to the approach we mentioned above. Results from FACS demonstrated that Cal27 cells promoted the membrane expression of CD163 and CD206 in PBMs (Supplementary Figure S2A). The mRNA expression of M2 phenotypic markers Arg1, Fizz1, Mgl1 and Mgl2 was also significantly increased in these primary macrophages when co-cultured with Cal27 cells (Supplementary Figure S2B). Moreover, RS504393 remarkably reduced the membrane expression of CD163 and CD206, as well as the mRNA expression of these M2 phenotypic cytokines in the PBMs under co-culture conditions (Supplementary Figure S2). Taken together, these results suggested that HNSCC cells promoted the differentiation and polarization of monocytes into M2- polarized macrophages by secreting CCL2.

### Macrophages promote the migration and invasion of HNSCC cells

**Figure 3:**
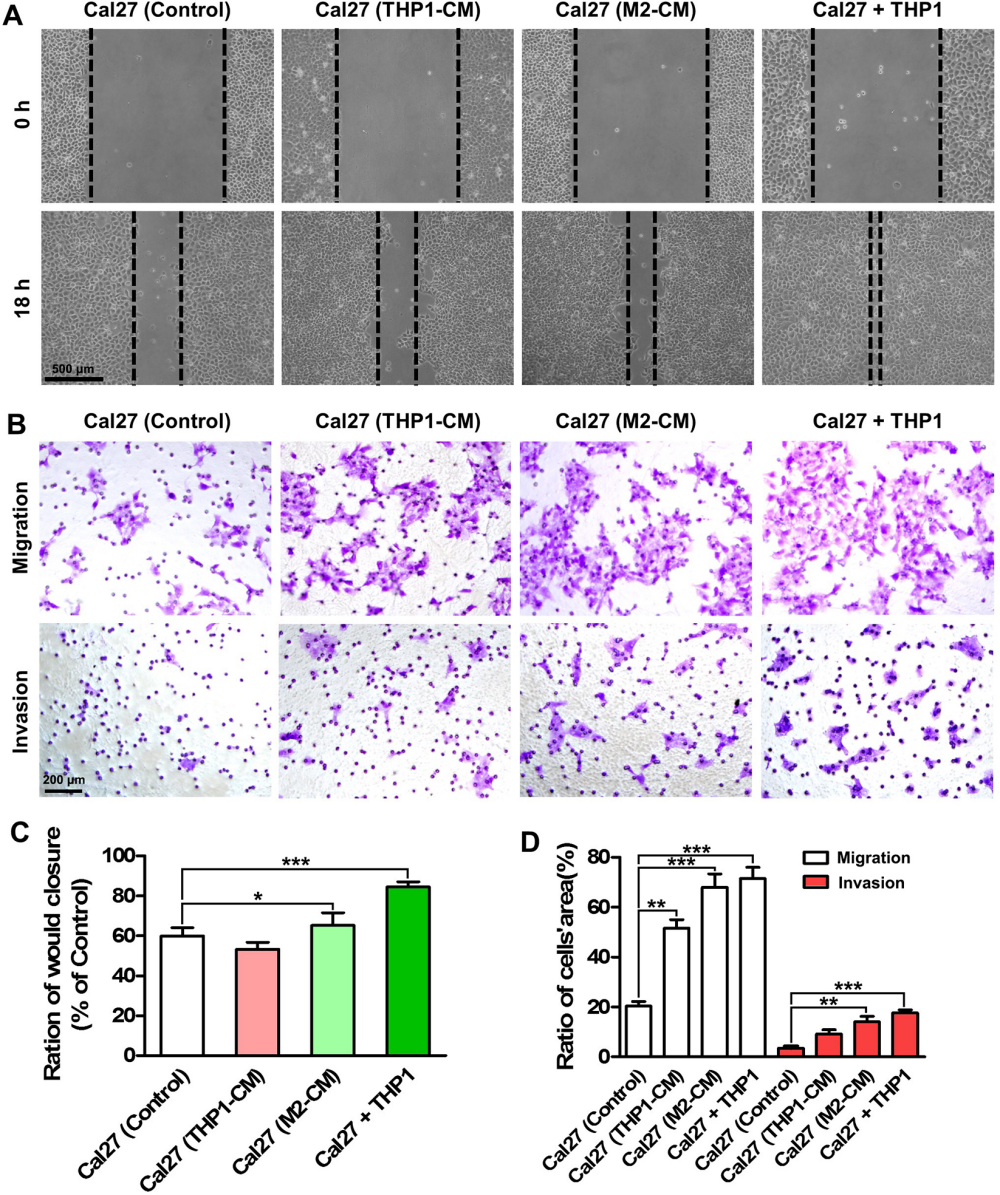
Co-culture with THP1 cells promotes the migration and invasion of HNSCC cells (**A**) Wound healing assays indicated the enhanced migration ability of Cal27 cells when co-cultured with THP1 cells. (**B**) The enhanced migration and invasion abilities of Cal27 cells in the co-culture system were determined by transwell chamber assays. (**C**) Quantitative analysis of transwell chamber assays. (**D**) Quantitative analysis of transwell chamber assays. The conditioned medium harvested from THP1 (THP1-CM) and induced M2-polarized macrophages (M2-CM) were used as a control. *P < 0.05, **P < 0.01, ***P < 0.001.

We next explored whether the interactions between cancer cells and macrophages contributed to the progression of HNSCC, especially to the invasion and metastasis. Thus, wound healing assays and transwell chamber assays were performed. As shown in Figure 3A and 3B, Cal27 cells in the direct co-culture system displayed the highest motility compared with those were treated with THP1 conditioned medium (THP1-CM) and M2- polarized macrophages conditioned medium (M2-CM). In addition, our data revealed that the co-cultured Cal27 cells held the highest invasive ability, even higher than the Cal27 cells treated with M2-CM. The quantitative data was presented in Figure 3C and 3D. The similar results were also found in the wound healing assays and transwell chamber assays using FaDu cells (Supplementary Figure S3). Intriguingly, we found that in the wound healing assays, most macrophages were located in the margins of the scratches, indicating that macrophages might lead HNSCC cells to migrate. Moreover, our results evidenced that suppression of CCL2/CCR2 axis using RS504393 alleviated the enhanced motility of Cal27 cells (Supplementary Figure S4). All these data indicated that macrophages in the co-culture system promoted the migration and invasion of HNSCC cells, thus contributing to the local invasion and distant metastasis of HNSCC.

### Macrophages enhance the invadopodia formation in HNSCC cells

**Figure 4:**
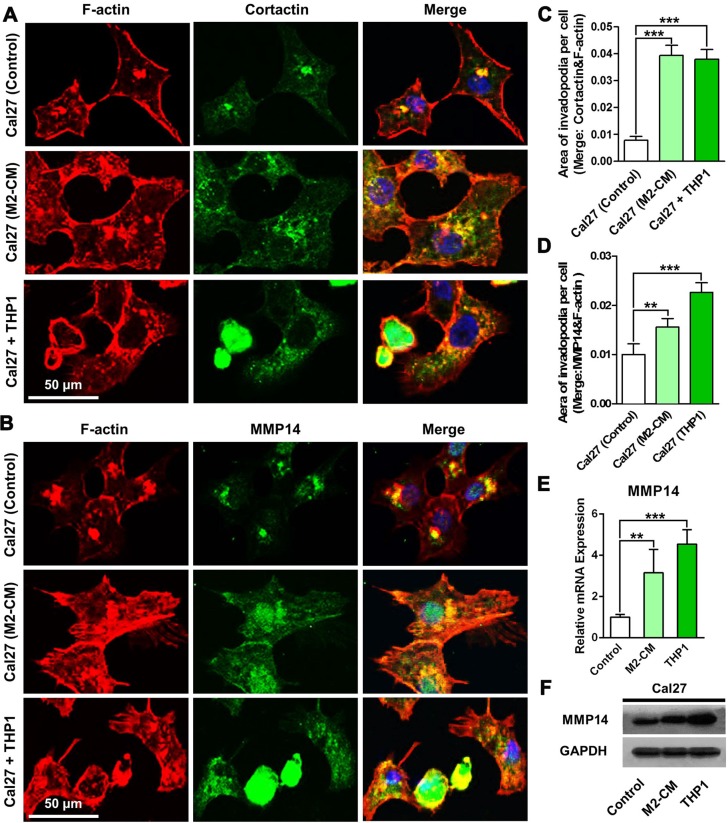
Co-culture with THP1 cells enhances the formation of invadopodia and the ECM degradation ability of HNSCC cells Cal27 cells alone or with THP1 cells were seeded on the gelatin coated coverslips. After 24 h, coverslips were harvested and stained with rhodamine-phalloidin and invadopodia biomarkers. (**A**) Co-culture with THP1 increased the Cortactin and F-actin positive invadopodia formation in Cal27 cells. (**B**) Co-culture with THP1 increased the MMP14 and F-actin positive invadopodia formation in Cal27 cells. (**C**) Quantitative analysis of the area of cortactin positive invadopodia per Cal27 cells. (**D**) Quantitative analysis of the area of MMP14 positive invadopodia per Cal27 cells. (**E**) The mRNA expression of MMP14 in the Cal27 cells were measured by qPCR. (**F**) The protein expression of MMP14 in the Cal27 cells were analyzed by western blots.

Accumulating research revealed invadopodia played important roles during cell migration and invasion [11, 17]. Thus invadopodia formation in HNSCC cells under co-culture conditions were analyzed. Co-localization of F-actin and cortactin in Cal27 cells proved invadopodia formation in the gelatin-coated coverslips. The increased yellow dots in the Cal27 cells co-cultured with THP1 suggested invadopodia were enhanced after interactions with macrophages (Figure 4A). By counting the area of the yellow dots per cell, we quantitatively analyzed the invadopodia formation. And we found that invadopodia were increased almost three times in the Cal27 cells that were directly co-cultured with THP1, similar with the cancer cells treated with M2-CM (Figure 4B). Moreover, co-localization of F-actin and MMP14 in Cal27 cells suggested the assembly of functional invadopodia, which took the responsibility to degrade ECM (Figure 4C). The quantitative data indicated that the functional invadopodia in Cal27 cells in the direct co-culture system were increased, as twice as that in the Cal27 cells in control group (Figure 4D). Moreover, mRNA and protein expression of MMP14 in Cal27 cells was analyzed using qPCR and western blots, respectively. In the co-cultured Cal27 cells, MMP14 was significantly increased in both mRNA and protein levels (Figure 4E and 4F). The increased invadopodia formation was also found in FaDu cells when co-cultured with THP1 (Supplementary Figure S5). ECM degradation ability of Cal27 cells was then analyzed by seeding cells on the coverslips coated by Alexa405-labeled gelatin. Under a confocal laser scanning microscope, the increased area of the matrix degradation (black holes) could be observed, suggesting that the ECM degradation ability of Cal27 cells was significantly enhanced (Supplementary Figure S6). Of great interest, the macrophages formed gigantic black holes, even bigger than that formed by activated Cal27 cells (in co-culture or treated with M2-CM). More, RS504393 suppressed the invadopodia formation of Cal27 cells under co-culture conditions (Supplementary Figure S7). In summary, our results proved the interactions between macrophages and HNSCC cells dramatically enhanced the migration and invasion of the tumor cells by increasing the functional invadopodia formation. In the co-culture system, macrophages might also contribute to the invasion through their remarkable ECM degradation abilities.

### CCL2/EGF paracrine loop contributes to the aggressive behavior of HNSCC cells

**Figure 5:**
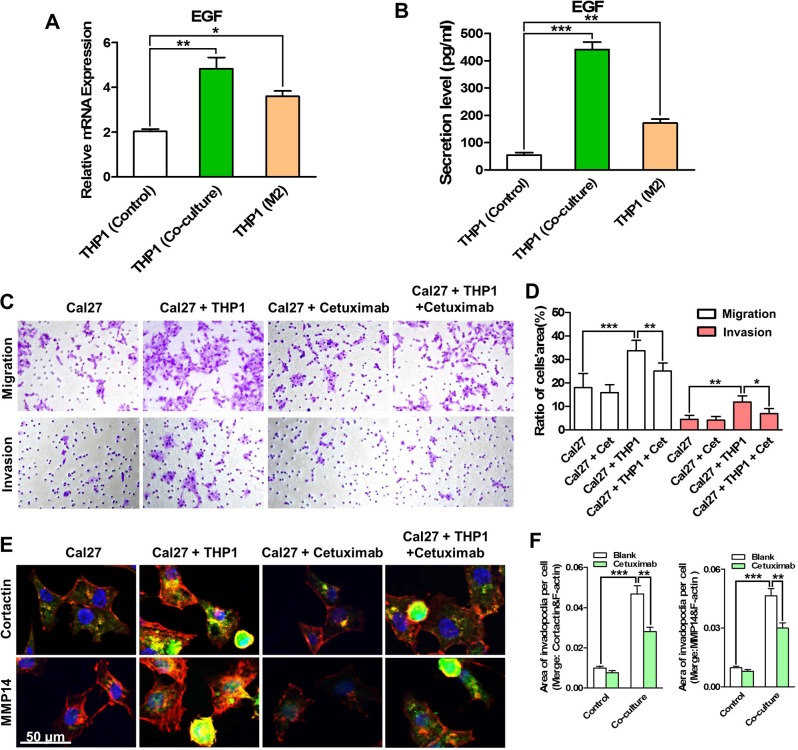
EGF derived by THP1 cells enhances the migration and invasion of HNSCC cells by increasing invadopodia formation (**A**) The EGF mRNA expression of THP1 in co-culture was measured. (**B**) The secretion level of EGF in the supernatant of the co-culture system was determined. THP1 alone or induced M2- polarized macrophages was used for comparison. (**C**) EGFR monoantibody Cetuximab suppressed the migration and invasion of Cal27 cells that were enhanced by the co-culture with THP1. (**D**) Quantitative analysis of transwell chamber assays. (**E**) Cetuximab reduced the invadopodia formation in Cal27 cells, especially that in co-cultured Cal27. (**F**) Quantitative analysis of the area of cortactin (left panel) and MMP14 (right panel) positive invadopodia per Cal27 cells. *P < 0.05, **P < 0.01, ***P < 0.001.

Previous studies reported that the invadopodia of tumor cells were regulated by EGF, a powerful growth factor that was highly expressed in TAMs [18, 19]. Thus, we measured the mRNA and protein expression of EGF in THP1 cells. As shown in Figure 5A and 5B, EGF was highly expressed in the THP1 cells that were co-cultured with Cal27 cells. Moreover, the protein level of EGF in the supernatant of the co-culture system was four times as high as that in the supernatant of pure THP1 cells. Cetuximab, an epidermal growth factor receptor (EGFR) monoclonal antibody that was widely used for cancer therapy, was exerted to block EGF/EGFR pathway. Our data showed the migration and invasion abilities of Cal27 cells were significantly impaired after cetuximab (10 μg/ml) treatment (Figure 5C and 5D, Supplementary Figure S8). More, cetuximab also reduced the invadopodia formation in the Cal27 cells under the co-culture conditions (Figure 5E and 5F) or treated with the M2- CM (Supplementary Figure S9).

**Figure 6:**
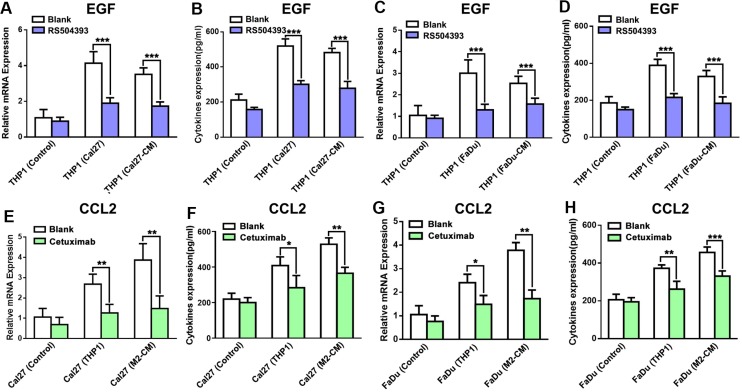
CCL2/CCR2 and EGF/EGFR axes act as a feedback paracrine loop between THP1 and HNSCC cells (**A**) RS504393 significantly reduced the EGF mRNA expression in THP1 cells that were co-cultured with Cal27. (**B**) RS504393 decreased the EGF level in the supernatant of the co-culture system with Cal27. (**C**) RS504393 significantly reduced the EGF mRNA expression in THP1 cells that were co-cultured with FaDu. (**D**) RS504393 decreased the EGF level in the supernatant of the co-culture system with FaDu. (**E**) Cetuximab reduced the CCL2 mRNA expression in Cal27 cells in the co-culture system. (**F**) Cetuximab alleviated the secretion level of CCL2 in the co-culture supernatant. (**G**) Cetuximab reduced the CCL2 mRNA expression in Cal27 cells in the co-culture system. (**H**) Cetuximab alleviated the secretion level of CCL2 in the co-culture supernatant. *P < 0.05, **P < 0.01, ***P < 0.001.

As CCL2 and EGF were up-regulated in HNSCC and THP1 cells in the co-culture system, it was reasonable to explore whether these two factors were regulated by each other. The mRNA and protein expression levels of EGF and CCL2 in the co-culture system were then analyzed. Results demonstrated RS504393 suppressed the expression of EGF in THP1 cells in both mRNA and protein levels under the co-culture conditions (Figure 6A–6D). On the other hand, cetuximab repressed the expression of CCL2 in Cal27 and FaDu cells that were co-cultured with THP1 cells (Figure 6E–6H). The similar results were also found under the indirect co-culture conditions. RS504393 significantly suppressed the increased expression of EGF in THP1 cells treated with Cal27 or FaDu conditioned medium (Cal27-CM; FaDu-CM), and cetuximab decreased the expression of CCL2 in Cal27 and FaDu cells that were treated with M2-CM. Taken together, these data suggested that monocytes that were co-cultured with HNSCC cells expressed and released EGF, which enhanced the migration and invasion of HNSCC cells by enhancing invadopodia formation. More importantly, a positive paracrine feedback loop of CCL2/EGF between macrophages and HNSCC cells was formed in the co-culture system.

### Curcumin blocks CCL2/EGF loop and inhibits malignant progression of HNSCC

**Figure 7:**
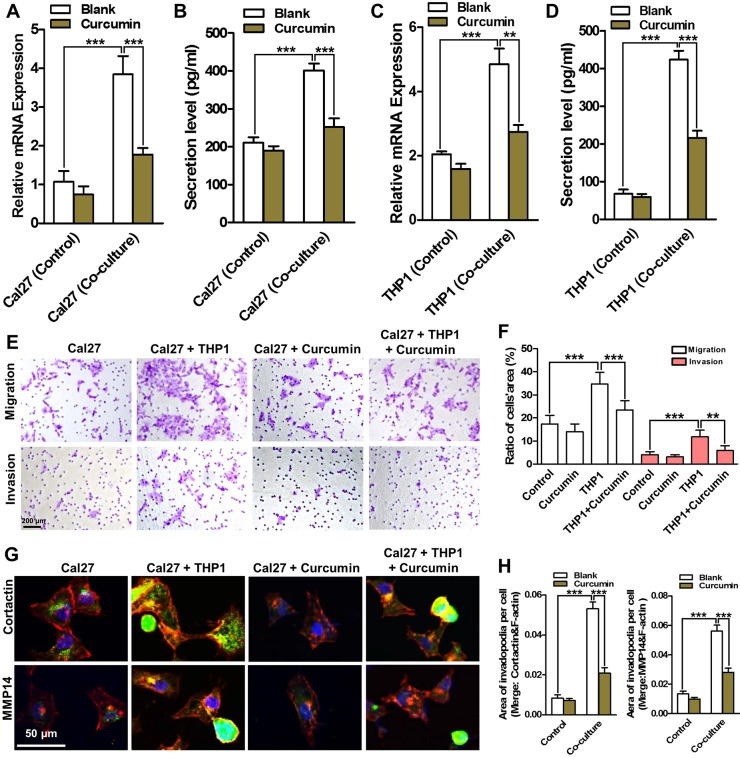
Curcumin inhibits the aggressive behaviors of HNSCC cells by breaking the CCL2/EGF paracrine loop Curcumin reduced the mRNA (**A**) and protein (**B**) expression levels of CCL2 in co-cultured Cal27, and decreased the expression of mRNA (**C**) and protein (**D**) in co-cultured THP1 cells. (**E**) Curcumin suppressed the migration and invasion of Cal27 cells in co-culture. (**F**) Quantitative analysis of transwell chamber assays. (**G**) Curcumin reduced the cortactin and MMP14 positive invadopodia formation in the co-cultured Cal27 cells. (**H**) Quantitative analysis of the area of cortactin (left panel) and MMP14 (right panel) positive invadopodia per Cal27 cells. *P < 0.05, **P < 0.01, ***P < 0.001.

Curcumin is a phenolic compound derived from *Curcuma longa*, showing great therapeutic potentials in both inflammatory diseases and malignancies [20]. We supposed curcumin might be able to block the crosstalk between monocytes and malignant cells. To determine the inhibitory effects on the cell proliferation of co-cultured THP1 and Cal27 cells, curcumin with different concentrations was added to the co-culture system. At a concentration of 10 μM or above, curcumin suppressed the proliferation of the cells under co-culture conditions obviously (data not shown). Thus, to avoid the effects of proliferation inhibition on the migration and invasion abilities of HNSCC cells, 5 μM curcumin was used in the following experiments. The expression of CCL2 and EGF in the co-culture system were firstly analyzed after curcumin As shown in Figure 7A and 7B, the expression of CCL2 was significantly reduced in Cal27 cells under co-culture conditions after curcumin treatment in both mRNA and protein levels, however, showed no obvious change in Cal27 cells cultured alone. On the other hand, the expression of EGF in THP1 cells co-cultured with Cal27 cells was also down-regulated in the presence of curcumin (Figure 7C and 7D). These results informed that curcumin might endow dual inhibitory effects in the therapeutics against HNSCC by targeting the CCL2/EGF positive feedback paracrine loop.

The migration and invasion abilities of Cal27 in the co-culture system after curcumin treatment were then analyzed. Curcumin (5 μM) significantly reduced the cell number on the bottom of transwell membrane in both migration and invasion assays, suggesting curcumin dramatically weakened the aggressive behavior of Cal27 cells under co-culture conditions (Figure 7E and 7F). Similar results were obtained from the wound healing assays (Supplementary Figure S10). Furthermore, our data revealed that curcumin only slightly decreased the invadopodia formation in Cal27 cells without any treatment, but significantly attenuated the functional invadopodia formation in the Cal27 cells under co-culture conditions (Figure 7G and 7H). Moreover, our results evidenced curcumin alleviated the ECM degradation in the co-culture system (Supplementary Figure S11). All these results demonstrated that curcumin significantly reduced the aggressive behaviors of HNSCC cells by targeting the CCL2/EGF positive feedback paracrine loop.

## DISCUSSION

The importance of the interactions between cancer cells and stromal cells during the progression of malignancies has been increasingly appreciated. As one of the most important stromal cell components, monocyte/macrophage has been attracting attentions since the early of this century. HNSCCs were closely related with chronic inflammation that was induced by smoking, injury or HPV infection [2]. Thus, we proposed that TAMs, the most important and abundant carcinoma associated inflammatory (CAI) cells, played prominent roles in HNSCCs. Previous studies including our recent work suggested the infiltration of macrophages was correlated with a poor outcome in HNSCC patients and could be used as a potentially useful prognostic marker [21, 22]. Despite numerous studies reported that monocytes/ macrophages were recruited in HNSCC tissues, the molecule mechanism by which macrophages or their precursors monocytes were recruited and activated is far from clear.

We thus established indirect and direct co-culture system between monocytes and HNSCC cells to study their interactions. As direct co-culture (but not indirect co-culture) between these two cell types might reflect the real situation in tumor tissues, it was established in our present study. The attachment of monocytes was considered as the marker of differentiation, and then the cells were sorted for the analysis of cytokines and membrane proteins expression to determine the polarization. Our screening data suggested that SDF-1α and CCL2 were increased significantly in HNSCC cells, but not GM-MSF and M-CSF as previously reported [23]. Blocking either of them disturbed the differentiation and polarization of monocytes. However, CCL2/CCR2 axis seemed much more critical during the process. CCL2, also named as monocyte chemotactic protein 1 (MCP1), was produced by stromal components in the tumor microenvironment and tumor cells. As a potent monocyte/macrophage chemotactic factor, CCL2 enhanced metastases in numerous tumors by recruiting macrophages [24, 25]. Recently, the elevated expression of CCL2 was also reported in HNSCC with a poor outcome in patients [26]. Blockade of CCL2/CCR2 axis in various tumors displayed remarkable therapeutic effects as the versatile functions of macrophages in tumor progression [10]. Of interest, we found that the corresponding receptors of SDF-1α and CCL2, CXCR4 and CCR2 were up-regulated in the THP1 cells, which might amplify the chemotaxis of monocytes into the tumor sites. Moreover, our data indicated that CCL2/CCR2 axis was involved in the M2 polarization of the attached macrophages.

TAMs, most of which were evidenced as M2- polarized macrophages, promoted malignant progression by secreting cytokines and growth factors [27]. In the present study, the up-regulation of EGF in the co-culture system was noticed. Blocking EGF/EGFR signaling pathway using cetuximab, we proved EGF was the foremost factor taking responsibility for the enhanced motility of HNSCC cells. Our above results reminded us that Cal27-derived CCL2 and THP1-derived EGF might form a positive feedback loop, thus promoting the differentiation of macrophages and the invasion of cancer cells. In breast cancers, it was reported that cancer cells activated macrophages to a TAM-like phenotype by producing GM-CSF, and reciprocally, CCL18 released from TAMs induced the epithelial mesenchymal transition (EMT) of breast cancer cells, thus forming a positive feedback loop. We then detected the CCL2 and EGF expression in the co-cultured Cal27 and THP1 cells after cexitumab and RS504393 treatment, respectively. We found blockade of EGF/EGFR axis reduced the expression of CCL2 in Cal27 cells, while suppression of CCL2/CCR2 axis decreased the expression of EGF in THP1 cells, suggesting that in HNSCC, the crosstalk between cancer cells and macrophages was also mediated by a positive feedback paracrine loop. Moreover, we noticed that the direct contact with THP1 was more powerful in promoting the motility of the cancer cells. Since most of THP1 cells were located on the surface of HSNCC cells, we proposed that the signals on the membrane of these two cells might interact and transmit additional signals for the enhanced migration and invasion. A previous research in breast cancers reported that the direct binding between tumor cells and macrophages mediated by CD11b/CD90 and Ephrin/EphA4 induced the EMT of breast cancer cells, thus establishing a cancer stem cell niche in a juxtacrine dependent manner [28]. It would be interesting and important to further uncover the physical interaction between HNSCC cells and macrophages.

Invadopodia were actin-rich cell membrane projections, by which invasive carcinoma cells penetrated the basement membrane and degraded ECM, promoting tumor local invasion. Moreover, recent studies uncovered that invadopodia were of importance for the intravasation of tumor cells [29]. Intravasation was a necessary step in the development of distant metastasis, through which the tumor cells entered the bloodstream [11]. Since our data proved that co-culture with THP1 cells enhanced the invasion ability of HSNCC cells, we further studied whether the formation of invadopodia in Cal27 cells was enhanced. The increased invadopodia in Cal27 cells was determined as the co-localized areas of cortactin and F-actin, as well as MMP14 and F-actin. The areas were enlarged dramatically in Cal27 cells in the co-culture system. Furthermore, degradation of the matrix was visualized by seeding cells on the Alexa405-gelatin coated coverslips. The results showed the enhanced matrix degradation ability of Cal27 cells under co-culture conditions. In addition, our data suggested that the Cal27 cells that were contacted macrophages directly showed more aggressive behaviors rather than those were treated with conditioned media of M2- polarized macrophages. These results were consistent with the finding in a recent study, in which the authors demonstrated the direct contact between macrophages and tumor cells induced RhoA activity in tumor cells, triggered the formation of invadopodia and then enabled tumor cells to break through the matrix barriers during transendothelial migration and metastasis [30]. Besides, in the gelatin degradation assays, we found that in comparison with Cal27 cells, THP1-derived macrophages presented much higher ECM degradation abilities as they formed bigger black holes in the Alexa405- labeled gelatin. Combined with the finding that macrophages were located on the margins of the scratches in the wound healing assays, we supposed that macrophages might act as the guide cells leading the migration and invasion of HNSCC cells, which was also partially proved in the Roh-Johnson *et al*., study [30]. However, whether the cancerous property of THP1 cells affects the ECM degradation ability should be further determined using primary macrophages.

Curcumin was widely studied in both inflammation-related diseases and malignancies. Therefore, we supposed it might be able to block the interactions between macrophages and HNSCC cells. As expected, curcumin broke the paracrine loop as it not only suppressed the expression of EGF in the macrophages but also inhibited the up-regulation of CCL2 in the HNSCC cells. Curcumin also alleviated the enhanced migration, invasion and ECM degradation abilities of the co-cultured Cal27 cells by reducing the invadopodia formation. Taken together, our present study uncovered a novel paracrine crosstalk between macrophages and tumor cells in HNSCC, and further highlighted the importance of macrophages in HNSCC cells migration and invasion by enhancing invadopodia formation. And most importantly, we found that curcumin, a normal and widely used natural drug, could alleviate the enhanced aggressive behaviors of HNSCC cells by blocking the CCL2/EGF positive feedback paracrine loop, shedding light on the great potentials in HNSCC treatment. Moreover, since the potent effects of the interaction between tumor cells and macrophage as we described in the present study, it was highly recommended to include the stromal cells when establishing *in vivo* models. And the paracrine loop we here reported also should be verified in an immunocompetent HNSCC mice model.

## MATERIALS AND METHODS

### Regents and antibodies

Dulbecco’s modified Eagle’s medium (DMEM), Roswell Park Memorial Institute medium (RPMI-1640), fetal bovine serum (FBS), penicillin, and streptomycin were obtained from GIBCO (Carlsbad, CA, USA). Dimethylsulfoxide (DMSO), Hoechst 33258 were purchased from Sigma-Aldrich (St. Louis, MO, USA). Carboxyfluorescein diacetate, succinimidyl ester (CFDA-SE) was purchased from Beyotime Institute of Biotechnology. Cetuximab was purchased from Merck (Darmstadt, Germany). AMD3100 and RS504393 were purchased from R&D Systems (Minneapolis, MN). Curcumin was purchased from Sigma-Aldrich (St. Louis, MO, USA). Rabbit polyclonal anti-cortactin antibodies, mouse monoclonal anti-MMP14 antibodies were purchased from Santa Cruz Biotechnology, Inc. (Santa Cruz, CA). Alexa Fluor 568-conjugated phalloidin and secondary anti-mouse or anti-rabbit antibodies conjugated with Alexa Fluor 488 or Alexa Fluor 568 were purchased from Invitrogen. Recombinant LPS, IL-4, IL13, IFN-γ was purchased from Peprotech. All other chemicals were classified as analytical grade reagents.

### Cell lines and culture conditions

THP1 (Human acute monocytic leukemia cell line) and Cal27 and FaDu (HNSCC cell lines) were obtained from the China Center for Type Culture Collection (CCTCC, Wuhan). THP1 was maintained in RPMI-1640, and Cal27 and FaDu were maintained in DMEM, supplemented with 10% FBS, 100 U/ml penicillin, and 100 mg/ml streptomycin. Cells were incubated in a humidified atmosphere of 95% air and 5% CO^2^ at 37°C. Cell volume was measured by the Vi-CELL cell viability analyzer (Beckman Coulter, Fullerton, CA, USA). The medium was changed three times a week and cells passaged when 70% confluence was reached.

### Induction of macrophages polarization and preparation of conditioned medium

THP1 cells were induced into M2- polarized macrophages by as previously reported. Briefly, THP1 cells were induced by phorbol 12-myristate 13-acetate (PMA) for 6h into macrophages, and then induced by IL-4 (20 ng/ml), IL-13 (20 ng/ml) for 18h into M2 macrophages. The culture medium was changed with FBS free medium and was collected as a conditional medium after further 24 h culture.

### Green fluorescent cells tracer technique

THP1 cells were centrifuged and suspended in the culture medium with 5% free FBS and green fluorescent dye CFDA-SE (2 μl/ml), incubating at 37°C for 15 min. Then the cells were centrifuged and suspended in the medium with 10% FBS, which incubated at 37°C for 30 min. Finally, the cells were rinsed with PBS twice.

### Adhesion assays

THP1 cells were stained by CFDA-SE and counted by the Vi-CELL cell viability analyzer. First, Cal27 cells (5 × 10^5^) were seeded in the six-well plate for 6 h. After Cal27 cells attachment, THP1 cells were seeded at the ratio of 1:1 in DMEM medium supplemented with or without RS504393, the group without Cal27 seeded as a control group. Cells were allowed to attach for 2 hours where after the wells were washed with PBS and the non-adherent cells of THP1 were collected and counted, then the adherent cells of THP1 were observed by microscope.

### Indirect and direct co-culture

The conditional medium (CM) was collected from Cal27, THP1 and M2- macrophages as described above. For indirect co-culture, Cal27-CM was put into six-well plates which seeded with THP1 or THP1 induced by PMA, and THP1-CM or M2-CM were put into 6 well plates which seeded with Cal27. For direct co-culture, THP1 cells were stained by CFDA-SE and seeded with Cal27 cells at a ratio of 1:1 in monolayer culture for 24 h.

### Fluorescence-activated cell sorting (FACS)

After establishing direct co-culture for 24 h, RS504393 (CCR2 inhibitor) and Cetuximab (EGFR inhibitor) were added into the co-culture system. Control group was performed in the same way and received the same medium but without medicine. After 24 h, the co-culture system was sorting by BD FACS III according to green fluorescence and size of cells. For flow cytometric analysis, the sorted THP1 cells were harvested and suspended in ice-cold PBS containing 3% BSA (Sigma). 5 × 10^5^ cells/tubes in 100 μl were prepared and incubated for 30 min. Cells were washed and incubated with PE anti-human CD163 and Alexa Fluor^®^ 647 anti-human CD206, as well as corresponding isotype antibodies for 30 min in dark. After washed 3 times, cells were analyzed on FACS Calibur flow cytometer (BD Biosciences) using Cell Quest software.

### MTT proliferation assays

Cal27 and THP1 were seeded at 5 × 10^3^ cells/well in a 96-well plate at a ratio of 1:1, which were starved of growth factors overnight by replacing their growth medium with DMEM (serum-free). Cells were incubated by curcumin with different concentrations (0, 5, 10, 20, 40 and 80 μM) as single stimuli. All conditions were tested in triplicate or quadruplicate. After 24 h, cell samples were incubated with 5 mg/ml MTT (Sigma) for 4 h. After removal of the MTT solution, formazan crystals were dissolved in dimethyl sulfoxide (DMSO). The absorbance was measured at a wavelength of 570 nm according to the manufacturer’s protocol.

### Quantitative real-time RT-PCR

Quantitative real-time RT-PCR (qPCR) was performed to evaluate the mRNA expression levels of macrophages-related genes, growth factors, chemokine and chemokine receptor in Cal27 and THP1 cells. Total RNA was isolated with TRIzol Reagent (Invitrogen). Aliquots (1 mg) of RNA were reverse transcribed to cDNA (20 μl) with oligo(dT) and M-MuLV reverse transcriptase (Fermentas, Glen Burnie, MD). One-fifth of the cDNA was used as a template for PCR using SYBR Premix ExTaq^TM^ (Perfect Real Time) kit (Takara, Kyoto, Japan) in an ABI 7500 Real-Time PCR System (Applied Biosystems, Foster City, CA). 18s rRNA was selected as an internal control for each experiment. The primer sequences designed for qPCR were presented in Supplementary Materials (Supplementary Table S1).

### Chemotactic migration and matrigel invasion assays

For detection of tumor cells migration and invasion, transwell boyden chambers (Corning, NY, USA) with polycarbonate filter (pore size of 8 μm) were used. Matrigel was diluted to 200 μg/mL and applied to the top side of the filter in cell invasion assays, whereas in cell migration assays, the filter was not coated. Cal27 cells were seeded on the upper chamber at a density of 1 × 10^5^ cells/well in 200μL of serum-deprived medium; meanwhile, conditioned medium was applied to the lower chamber as chemoattractant (indirect co-culture). Cal27 cells and THP1 cells (0.5 × 10^5^ cells each) were seeded on the upper chamber (direct co-culture). After 24 h incubation, the cells on the upper surface of the membrane were carefully removed by scrubbing with a cotton swab. Then, the membranes were fixed with 4% formaldehyde for 10 min, stained with 1% crystal violet for 5 min, and finally photographed and quantified under a phase microscope.

### Would healing assays

Cal27 cells (1 × 10^6^/well) or Cal27 and THP1 cells (0.5 × 10^5^ cells each) were plated in 6-well plates and were grown to 80–90% confluence. Then, the monolayer of cells was scraped with a standard 200 μL sterile micropipette tip to create a denuded gap of constant width. The cells were subsequently washed with PBS and then exposed to conditioned medium or serum-free medium for 24 h. At the end of the exposure, the cells migrated in the gap were observed and counted under a phase microscope.

### Invadopodia and extracellular matrix (ECM) degradation assays

Briefly, 0.2% gelatin in PBS was dropped on the coverslips that were pretreated with 0.5% glutaraldehyde for 15 min at room temperature. Then cells were seeded on gelatin matrix coated coverslips in 24-well plates and grown for 24 h. Alexa Fluor^®^ 408 Protein Labeling Kit was used to label gelatin for extracellular matrix degradation assays. The cells were grown on glass coverslips with indicated treatment. Then, cells were washed with PBS, fixed in paraformaldehyde (PFA) at 37°C for 30 min, washed with PBS for three times and blocked with 10% non-immune goat serum for 1 h at room temperature. After that, cells were incubated with the primary antibody including cortactin and MMP14 at a dilution of 1:200 overnight at 4°C, followed by incubation with DyLight 488-conjugated secondary antibody (1:400) and rhodamine-conjugated phalloidin (1:200) for 1 h at room temperature. The nuclei were stained with DAPI, and the coverslips were mounted on a microscope slide with embedding medium (Invitrogen, Carlsbad, CA, USA). The cells were observed and photographed with a confocal microscope. Olympus FV1000 confocal laser scanning microscope and Leica SP8 confocal microscope were used. Co-localization parameters were measured by Leica Microsystems software Quantitative analysis of the number of invadopodia per cell, the area of individual invadopodia formed by cells was performed using Image pro plus software. For each condition, only invadopodia positive for F-actin and cortactin or MMP14 were scored and 10 cells per each slide were analyzed.

### Enzyme-linked immunosorbent assay (ELISA)

ELISA kits for EGF and CCL2 were used (R&D Systems, Minneapolis, MN). Concentrations of human EGF and CCL2 in the culture supernatant were measured by ELISA following kit instructions. Briefly,100 μL of the samples were loaded on the plates and incubated for 2 h at room temperature. After the plates were washed with wash buffer (0.05% Tween20 in PBS), samples were incubated with detection antibody for 2 h at room temperature. Immunoreactivity was determined by adding substrate solution and absorbance (450 nm and 570 nm) was determined by Vmax Kinetic microplate reader (Molecular Devices, Sunnyvale, CA).

### Statistical analysis

All data were presented as mean ± SEM of three independent or more experiments. One/two-way analysis of variance, Student-Newman-Keuls were performed for statistical analysis. Mean ± SEM with a difference of *P* < 0.05 was considered statistically significant.

## SUPPLEMENTARY MATERIALS TABLE AND FIGURES


