# Supplementary material for: CCL2/EGF positive feedback loop between cancer cells and macrophages promotes cell migration and invasion in head and neck squamous cell carcinoma
Source: Oncotarget. 2016 Nov 23;7(52):87037–51. doi: 10.18632/oncotarget.13523 (PMC5349969; doi:10.18632/oncotarget.13523)
Supplement: Supplementary file 1 [file oncotarget-07-87037-s001.pdf]

CCL2/EGF positive feedback loop between cancer cells and macrophages promotes cell migration and invasion in head and neck squamous cell carcinoma

Supplementary Materials

MATERIALS AND METHODS

Isolation and culture of human peripheral blood monocytes

Human peripheral blood monocytes (PBMs) were isolated according to the previous studies (Delirezh N, 2013; Sørensen MG, 2007). The study was approved by the review board of the Ethics Committee of Hospital of Stomatology, Wuhan University. The procedures for human tissues were performed according to the National Institutes of Health guidelines. Briefly, 4ml of the human venous blood sample in heparinised vials was collected from the volunteers. Then the peripheral blood mononuclear cells (including monocytes and lymphocytes) were isolated by gradient centrifugation using Ficoll-Histophaque. After washing with PBS for twice, the samples were resuspended in 30 μl per 10^7 total cells according to the manufacture's instruction (Miltenyi Biotec). FcR blocking buffer was added. Then the cells were selected with the anti-CD14-coated magnetic microbeads. The isolated monocytes were used immediately after cell counting as we described in the manuscript.

REFERENCES

1. Delirezh N, Shojaeefar E, Parvin P, Asadi B. Comparison the effects of two monocyte isolation methods, plastic adherence and magnetic activated cell sorting methods, on phagocytic activity of generated dendritic cells. Cell J. 2013 Fall; 15:218–23.
2. Sørensen MG, Henriksen K, Schaller S, Henriksen DB, Nielsen FC, Dziegiel MH, Karsdal MA. Characterization of osteoclasts derived from CD14+ monocytes isolated from peripheral blood. J Bone Miner Metab. 2007; 25:36–45.

Supplementary Table S1: Summary of primers used in present study for real-time PCR analysis

Gene	Sequence (5′–3′)
Arg1	Forward: CCACAGTCTGGCAGTTGGAAG<br>Reverse: GGTTGTCAGGGGAGTGTTGATG
Fizz1	Forward: CCTGCTGGGATGACTGCTA<br>Reverse: TGGGTTCTCCACCTCTTCAT
Mgl1	Forward: CAGAATCGCTT AGCCAATGTGG<br>Reverse: TCCCAGTCCGTGTCCGAAC
Mgl2	Forward: TTCAAGAATTGGAGGCCACT<br>Reverse: CAGACATCGTCATTCCAACG
SDF-1α	Forward: TGCATCAGTGACGGTAAACCA<br>Reverse: CACAGTTTGGAGTGTTGAGGAT
CCL2	Forward: AAGATCTCAGTGCAGAGGCTCG<br>Reverse: TTGCTTGTCCAGGTGGTCCAT
CXCR4	Forward: AGCATGACGGACAAGTACC<br>Reverse: GATGATATGGACAGCCTTACAC
CCR2	Forward: TGCTCTAGAGAAGACAATAATATGTTACC<br>Reverse: ATAGCGGCCGCTTACAACCCAACCGAGACCT
EGF	Forward: TCATCTGCTCTAATGCAGGTACA<br>Reverse: GTTTCCACAGTAACACTTCCCA
18s rRNA	Forward: CGGCTACCACATCCAAGGAA<br>Reverse: GCTGGAATTACCGCGGCT

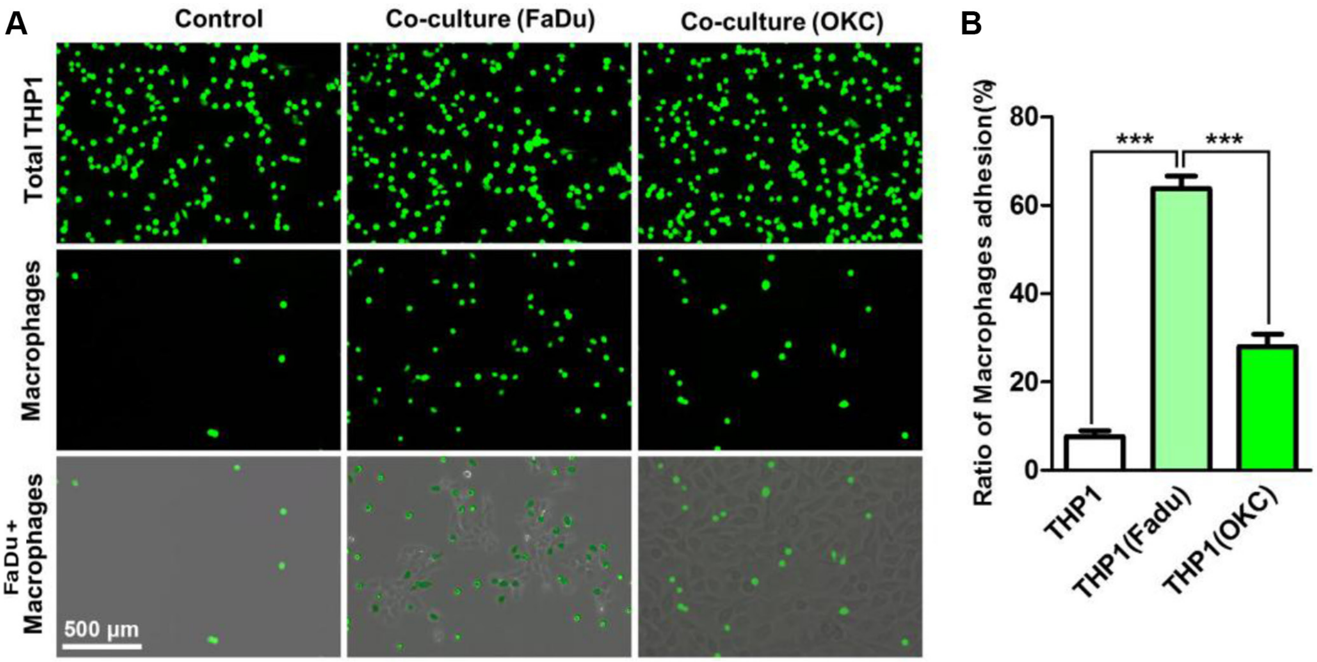

Supplementary Figure S1: FaDu cells promote the differentiation of THP1 cells. CFDA-SE labeled THP1 cells were added to the dishes in which FaDu cells were seeded for 6 h. After co-culture for 24 h, unattached THP1 cells were washed out. (**A**) FaDu cells promote the differentiation of THP1 cell into macrophages, most of which were attached on the cancer cells. (**B**) The ratio of attached cells to total added THP1 cells was calculated. ***$P < 0.001$.

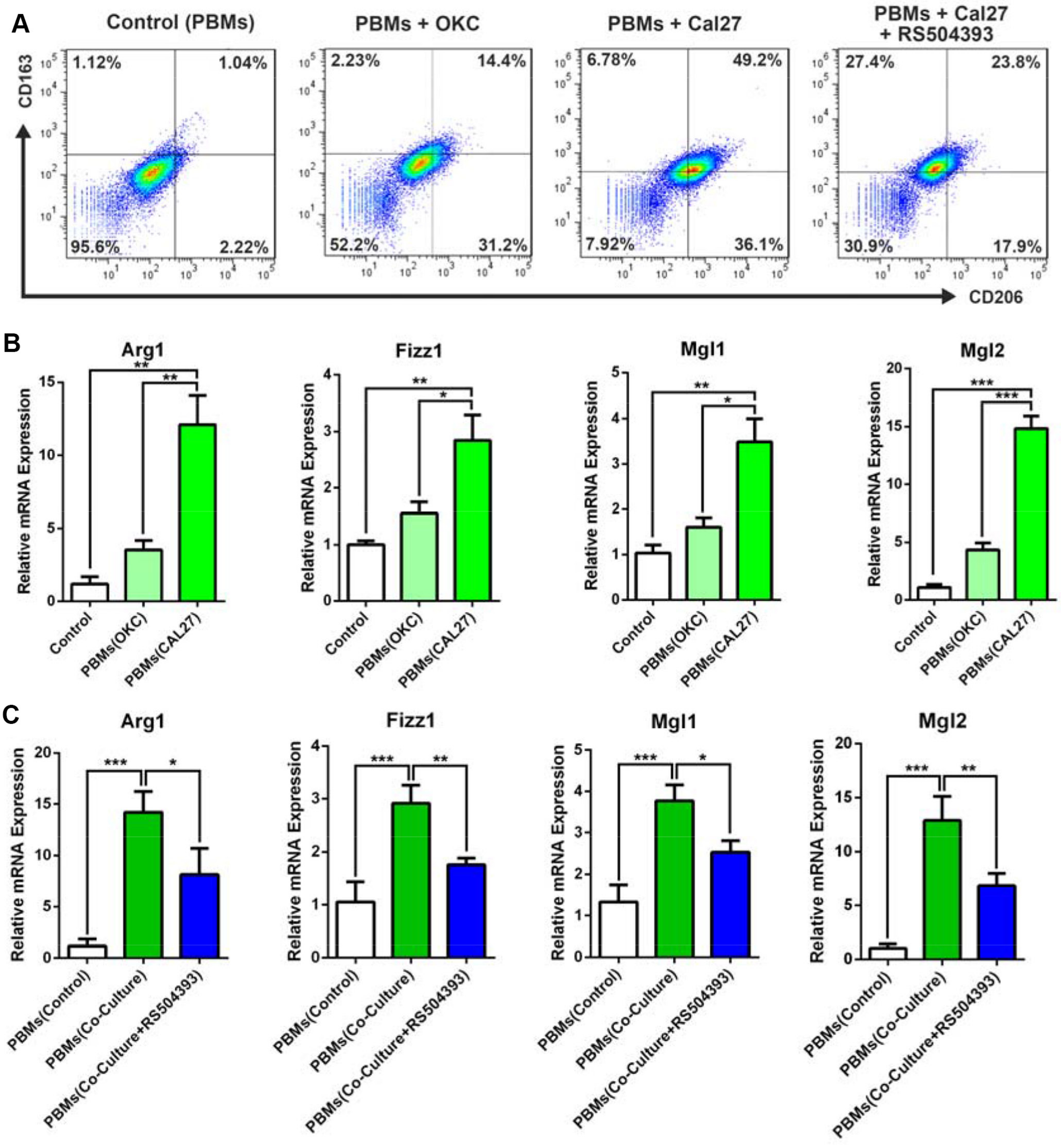

Supplementary Figure S2: Cal27 cells promote the M2- polarization of human peripheral blood monocytes in a CCL2 dependent manner. (**A**) The CD163 and CD206 double positive peripheral blood monocytes (PBMs) were quantitatively analyzed. (**B**) The mRNA expression of M2- polarized macrophage's markers Arg1, Fizz1, Mgl1 and Mgl2 in PBMs were analyzed. (**C**) The mRNA expression of Arg1, Fizz1, Mgl1 and Mgl2 in PBMs were analyzed after treatment of RS504393 (10 μM). *$P < 0.05$; **$P < 0.01$; ***.

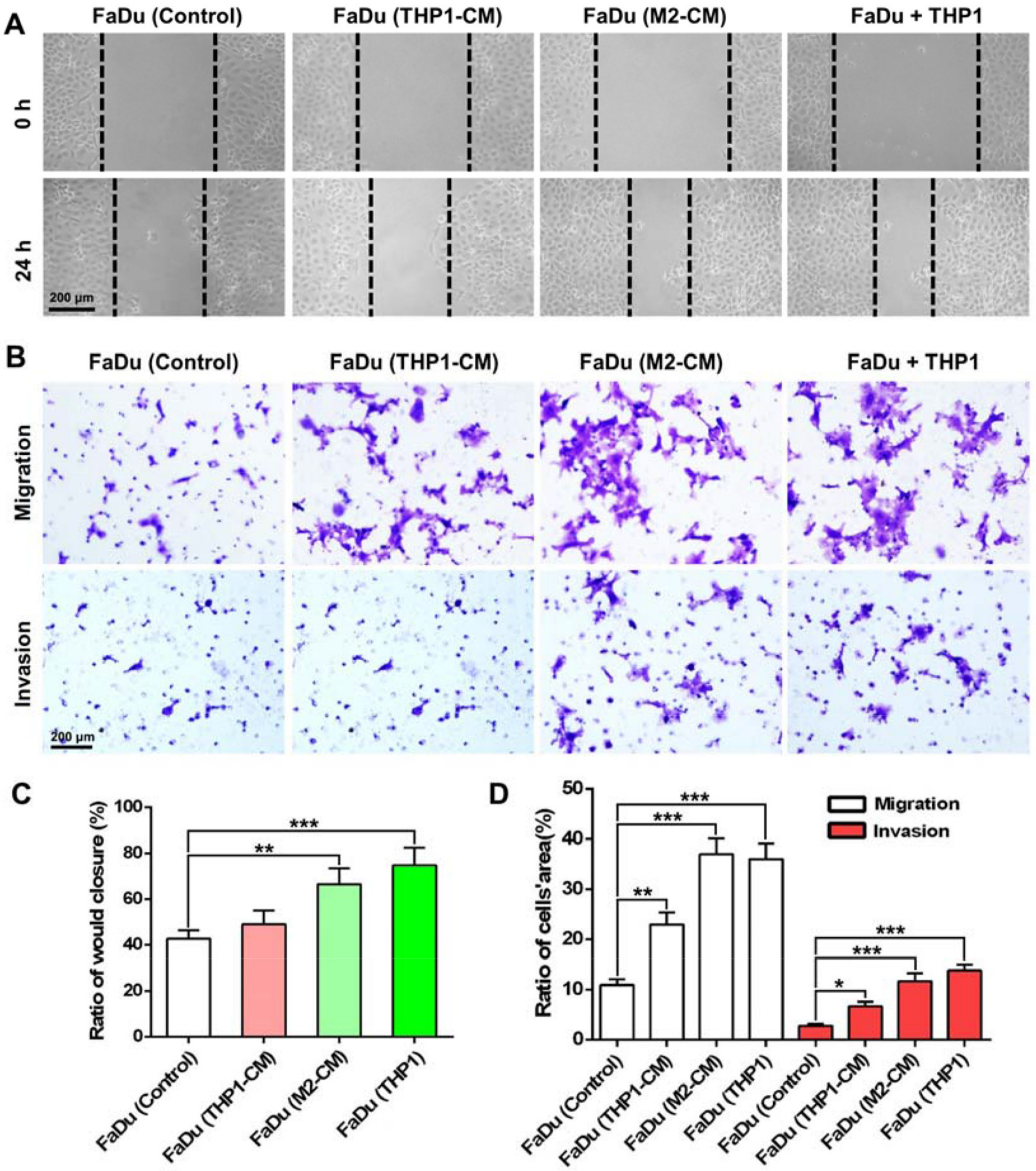

Supplementary Figure S3: Co-culture with THP1 cells promotes the migration and invasion of FaDu cells. (**A**) Wound healing assays indicated the enhanced migration ability of FaDu cells when co-cultured with THP1 cells. (**B**) The enhanced migration and invasion abilities of FaDu cells in the co-culture system were determined by transwell chamber assays. (**C**) Quantitative analysis of wound healing assays. (**D**) Quantitative analysis of transwell chamber assays. The conditioned medium harvested from THP1 (THP1-CM) and induced M2-polarized macrophages (M2-CM) were used as a control. $*P < 0.05$, $**P < 0.01$, $***P < 0.001$.

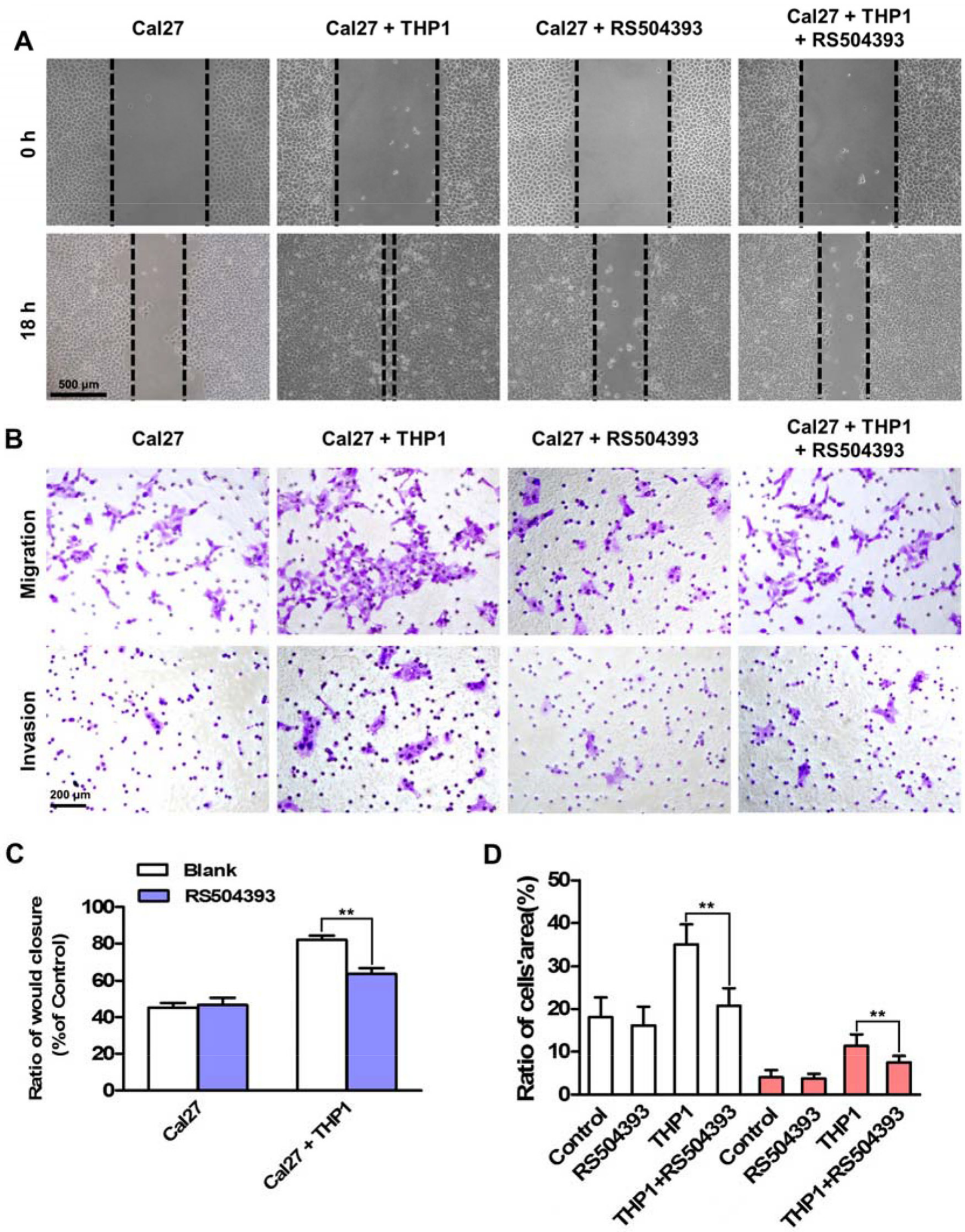

Supplementary Figure S4: RS504393 suppresses the migration and invasion of Cal27 in co-culture. (**A**) Wound healing assays indicated RS504393 suppressed the migration of Cal27 which was enhanced by co-culture with THP1 cells. (**B**) Transwell chamber assays determined RS504393 decreased the migrated and invasive cells in the co-culture system. (**C**) Quantitative analysis of wound healing assays. (**D**) Quantitative analysis of transwell chamber assays. **P* < 0.05; ***P* < 0.01; ****P* < 0.001.

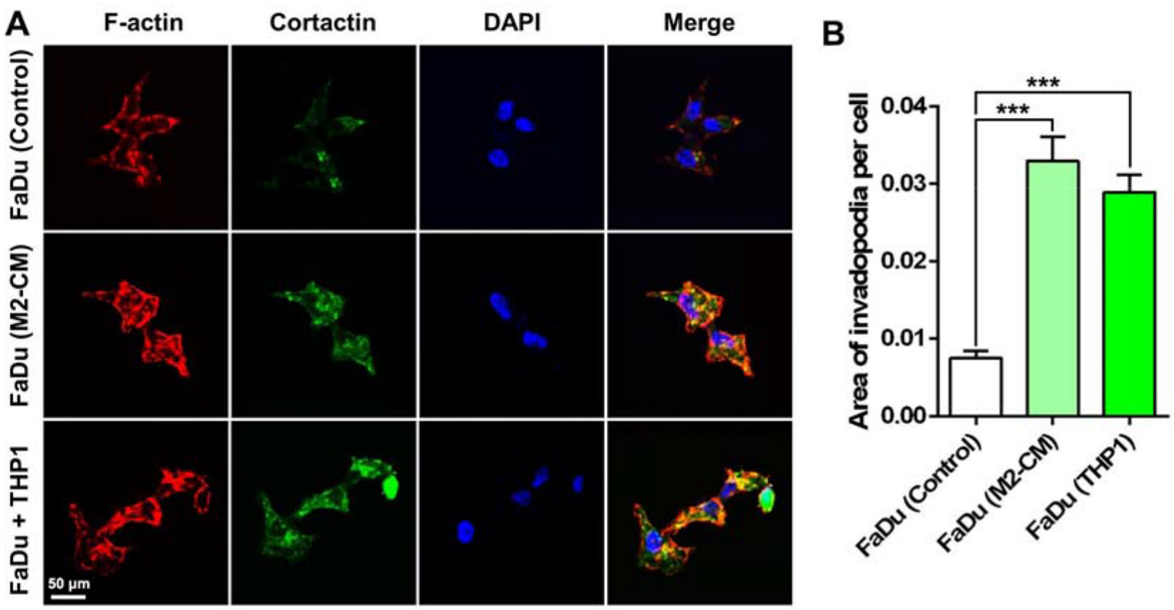

Supplementary Figure S5: Co-culture with THP1 cells enhances the formation of invadopodia in FaDu cells. FaDu cells alone or with THP1 cells were seeded on the gelatin-coated coverslips. After 24 h, coverslips were harvested and stained with rhodamine-phalloidin and invadopodia biomarkers. (**A**) Co-culture with THP1 increased the Cortactin and F-actin positive invadopodia formation in FaDu cells. (**B**) Quantitative analysis of the area of Cortactin positive invadopodia per FaDu cells. ***$P < 0.001$.

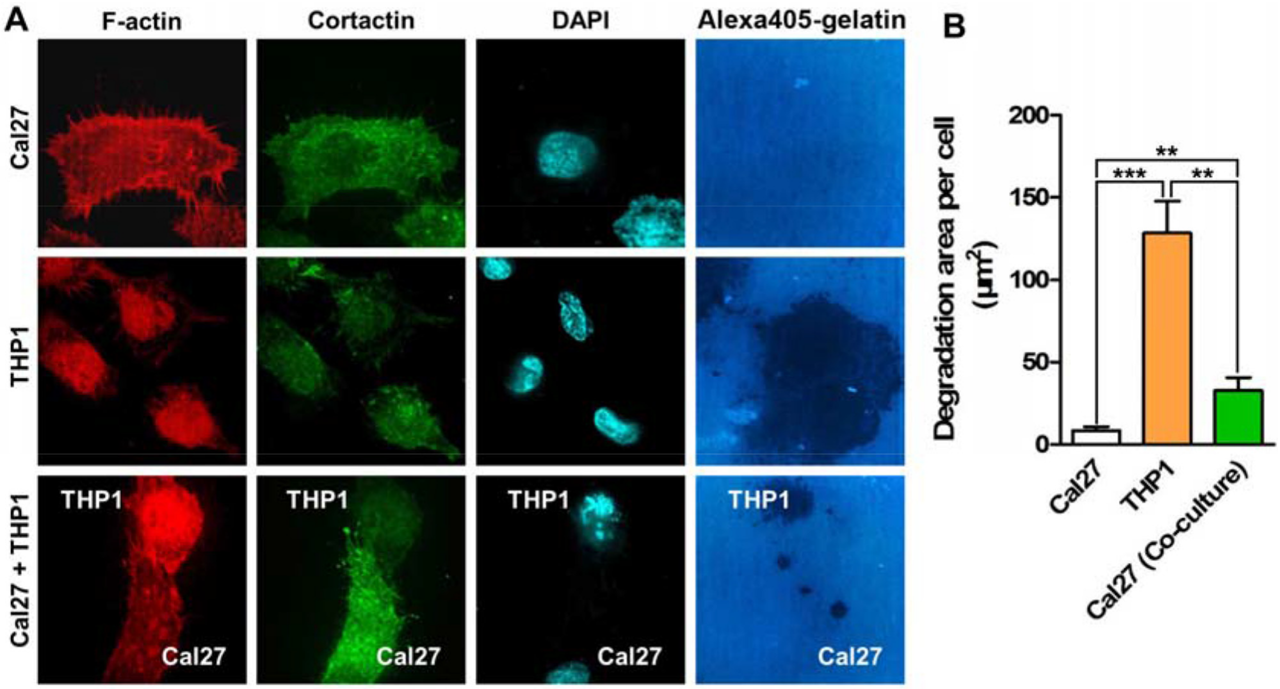

Supplementary Figure S6: Co-culture with THP1 cells enhances the extracellular matrix (ECM) degradation of Cal27 cells. (**A**) Four color immunofluorescence proved the formation of invadopodia by determining the co-localization of Cortactin and F-actin, and the ECM degradation by detecting the black holes of Alexa405 labeled gelatin. (**B**) Quantitative analysis of the ECM degradation ability of Cal27 cells by measuring the black holes' area per cell. **$P < 0.01$, ***$P < 0.001$.

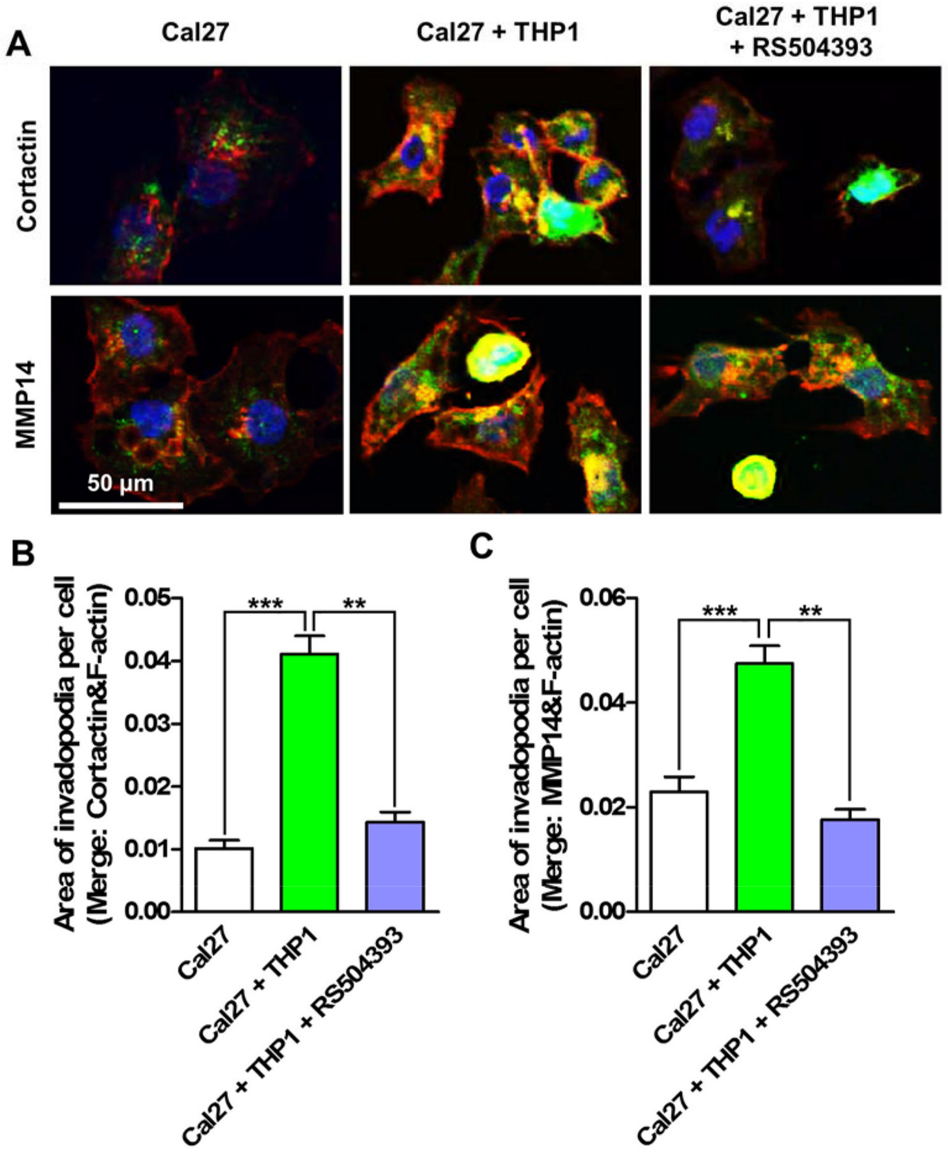

Supplementary Figure S7: RS504393 suppresses the formation of invadopodia in Cal27 cells co-cultured with THP1. (**A**) RS504393 decreased the Cortactin and F-actin positive and MMP14 and F-actin positive invadopodia formation in Cal27 cells that were co-cultured with THP1 cells. (**B**) Quantitative analysis of the area of Cortactin positive invadopodia per Cal27 cells. (**C**) Quantitative analysis of the area of MMP14 positive invadopodia per Cal27 cells. ***$P < 0.001$.

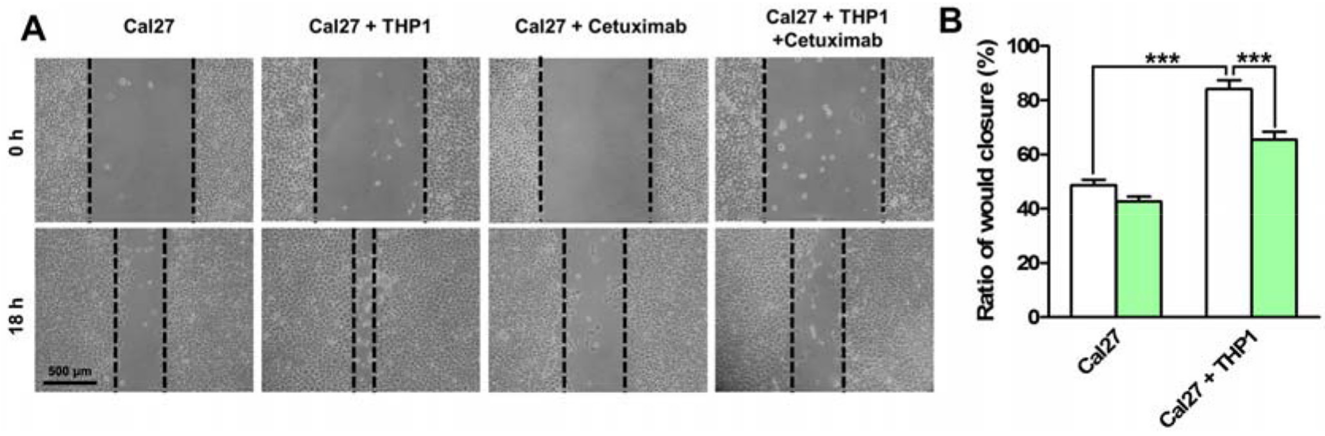

Supplementary Figure S8: Cetuximab alleviates the migration of Cal27 cells in co-cultured. (**A**) Wound healing assays indicated that Cetuximab decreased the migration of Cal27 cells that were co-cultured with THP1 cells. (**B**) Quantitative analysis of wound healing assays. ***$P < 0.001$.

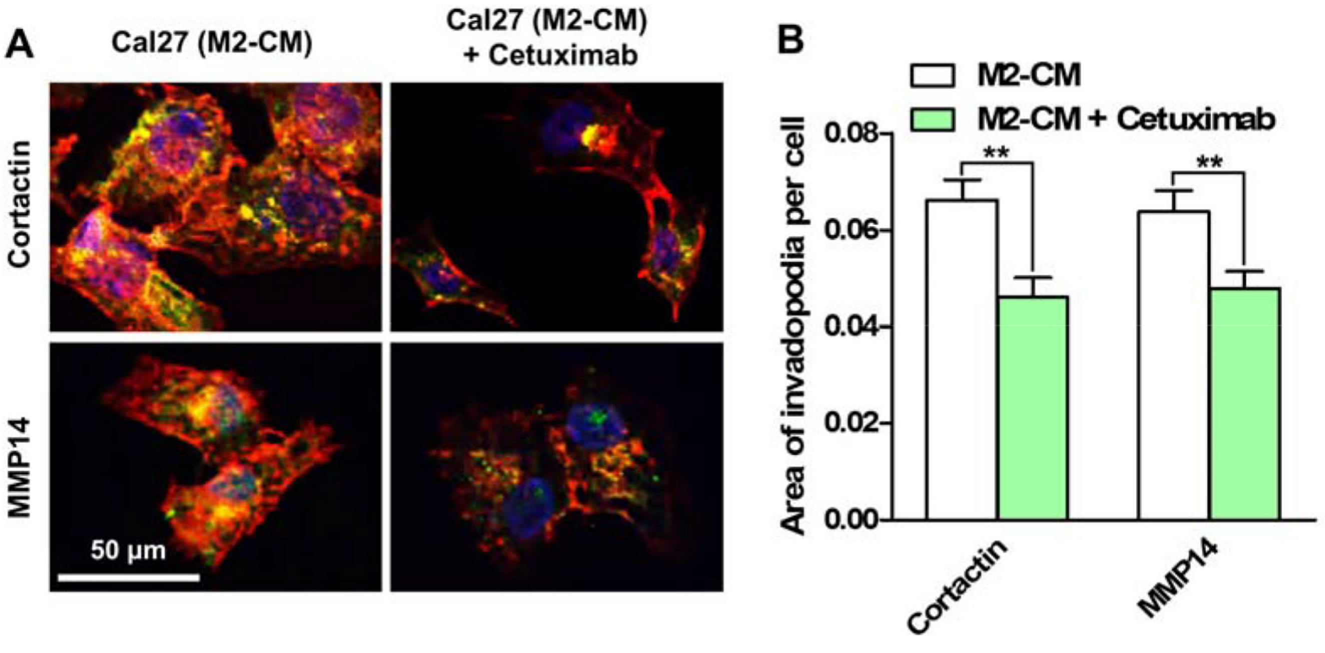

Supplementary Figure S9: Cetuximab suppresses the enhanced invadopodia formation in Cal27 cells treated with the conditioned medium of M2 macrophages (M2-CM). (**A**) Cetuximab suppressed the Cortactin/F-actin, and MMP14/F-actin positive invadopodia formation in Cal27 cells. (**B**) Quantitative analysis of the area of invadopodia per Cal27 cells. **$P < 0.01$.

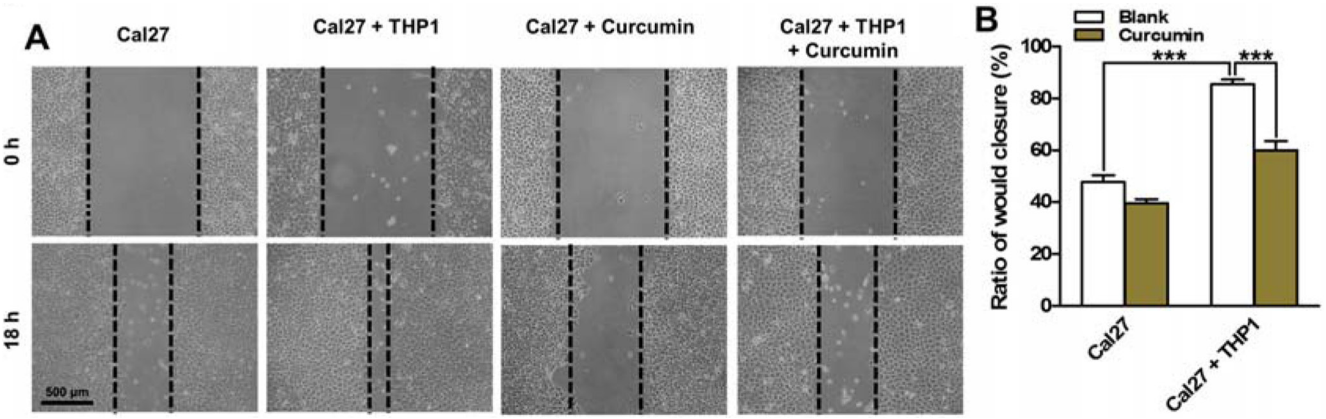

Supplementary Figure S10: Curcumin suppresses the migration of Cal27 cells in co-cultured. (**A**) Wound healing assays revealed that curcumin decreased the migration of Cal27 cells that were co-cultured with THP1 cells. (**B**) Quantitative analysis of wound healing assays. ***$P < 0.001$.

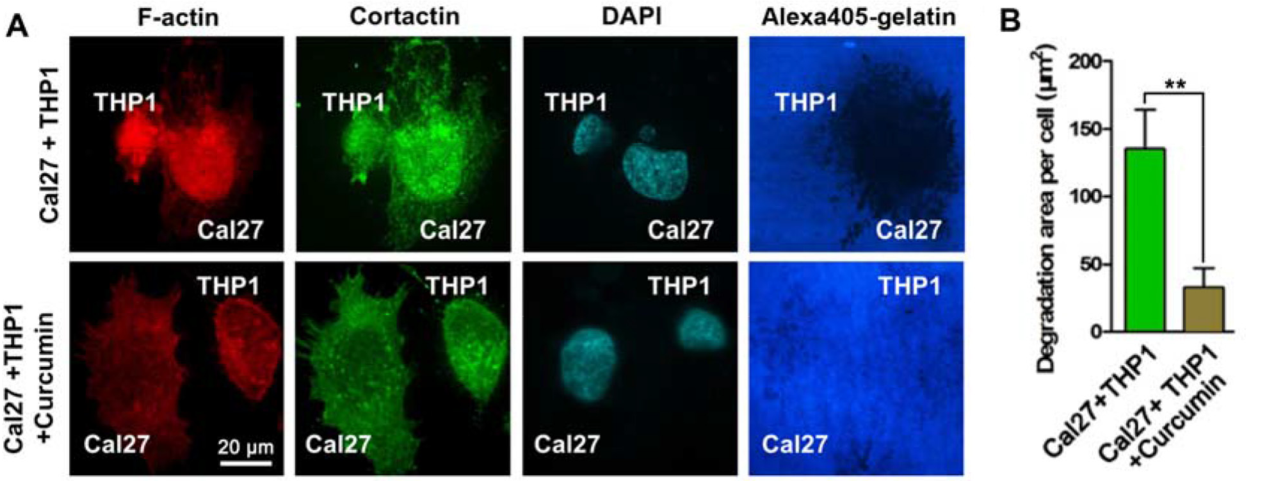

Supplementary Figure S11: Curcumin weakens the extracellular matrix degradation ability of Cal27 cells in co-culture. (**A**) Four color immunofluorescence suggested the formation of invadopodia by determining the co-localization of Cortactin and F-actin, and the ECM degradation by detecting the black holes of Alexa405 labeled gelatin. (**B**) Quantitative analysis of the ECM degradation ability of Cal27 cells by measuring the black holes' area per cell. **$P < 0.01$.
